# *Caenorhabditis elegans* Semi-Automated Liquid Screen Reveals a Specialized Role for the Chemotaxis Gene *cheB2* in *Pseudomonas aeruginosa* Virulence

**DOI:** 10.1371/journal.ppat.1000540

**Published:** 2009-08-07

**Authors:** Steven Garvis, Antje Munder, Geneviève Ball, Sophie de Bentzmann, Lutz Wiehlmann, Jonathan J. Ewbank, Burkhard Tümmler, Alain Filloux

**Affiliations:** 1 Laboratoire d'Ingénierie des Systèmes Macromoléculaires, UPR9027, Centre National de la Recherche Scientifique, IMM, Marseille, France; 2 Klinische Forschergruppe, Center of Biochemistry and Pediatrics, Hannover Medical School, Hannover, Germany; 3 Centre d'Immunologie de Marseille-Luminy, Université de la Méditerranée, Case 906, Marseille, France; 4 INSERM, U631, Marseille, France; 5 CNRS, UMR6102, Marseille, France; 6 Imperial College London, Centre for Molecular Microbiology and Infection, Division of Cell and Molecular Biology, South Kensington Campus, London, United Kingdom; Massachusetts General Hospital, United States of America

## Abstract

*Pseudomonas aeruginosa* is an opportunistic human pathogen that causes infections in a variety of animal and plant hosts. *Caenorhabditis elegans* is a simple model with which one can identify bacterial virulence genes. Previous studies with *C. elegans* have shown that depending on the growth medium, *P. aeruginosa* provokes different pathologies: slow or fast killing, lethal paralysis and red death. In this study, we developed a high-throughput semi-automated liquid-based assay such that an entire genome can readily be scanned for virulence genes in a short time period. We screened a 2,200-member STM mutant library generated in a cystic fibrosis airway *P. aeruginosa* isolate, TBCF10839. Twelve mutants were isolated each showing at least 70% attenuation in *C. elegans* killing. The selected mutants had insertions in regulatory genes, such as a histidine kinase sensor of two-component systems and a member of the AraC family, or in genes involved in adherence or chemotaxis. One mutant had an insertion in a *cheB* gene homologue, encoding a methylesterase involved in chemotaxis (CheB2). The *cheB2* mutant was tested in a murine lung infection model and found to have a highly attenuated virulence. The *cheB2* gene is part of the chemotactic gene cluster II, which was shown to be required for an optimal mobility *in vitro*. In *P. aeruginosa*, the main player in chemotaxis and mobility is the chemotactic gene cluster I, including *cheB1*. We show that, in contrast to the *cheB2* mutant, a *cheB1* mutant is not attenuated for virulence in *C. elegans* whereas in vitro motility and chemotaxis are severely impaired. We conclude that the virulence defect of the *cheB2* mutant is not linked with a global motility defect but that instead the *cheB2* gene is involved in a specific chemotactic response, which takes place during infection and is required for *P. aeruginosa* pathogenicity.

## Author Summary

The increase in hospital acquired and multi-drug resistant bacterial infections calls for an urgent development of new antimicrobials. As such, the identification and characterization of novel molecular targets involved in bacterial virulence has become a common goal for researchers. The use of non-mammalian hosts, such as the nematode *Caenorhabditis elegans*, is useful to accelerate this process. In our study, we developed a high-throughput screening method, which further facilitates the use of *C. elegans*, and allows the rapid screening of a large collection of bacterial mutants at the genomic scale. We have used *Pseudomonas aeruginosa*, a potent opportunistic pathogen, to perform this study. The screening of more than 2,000 mutant strains allowed the characterization of a mutant affected in the *cheB2* gene. Importantly, this mutant was shown to be impaired in a mouse model of infection, supporting that our new screen is a good model to identify virulence genes relevant for infection in mammals. The *cheB2* gene encodes a component of a chemotaxis pathway, which is likely involved in the perception of stimuli during the infection process, and allows an appropriate adaptive response for a successful infection. Our method could be applied to other bacterial pathogens and will help researchers discover candidate genes leading to the design of novel antimicrobials.

## Introduction

The ubiquitous Gram-negative bacterium *Pseudomonas aeruginosa* is an opportunistic pathogen able to infect a broad range of animals and plants hosts including humans. In the course of infection, *P. aeruginosa* adapts to changing environmental conditions and coordinates the production of molecular determinants involved in host colonization and virulence [1]. Among these are pili and flagella, which are required for attachment and spreading on surfaces [2],[3]. Also necessary are protein secretion systems [4] and toxins required for cytotoxicity and survival within the host. One powerful approach to dissect the interaction between pathogen and host is the use of simple infection models. It has been demonstrated that the nematode *Caenorhabditis elegans* can be an effective model for studying virulence mechanisms used by a variety of bacterial pathogens [5],[6]. *P. aeruginosa* is capable of killing *C. elegans* in several distinct ways. When the *P. aeruginosa* strain PA14 is cultured on a high-osmolarity peptone-glucose-sorbitol medium (PGS), worms succumb to intoxication termed “fast killing”, as the exposed worms die within hours [7]. Nematodes exposed to PA14 grown on nematode growth media (NGM), succumb to “slow killing”. In this case the bacteria colonize the gut and the infected worms die over a number of days rather than hours [8]. The *P. aeruginosa* isolate PAO1 cultured on Brain Heart Infusion (BHI) agar induces worm paralysis and death within hours [9]. Finally, *C. elegans* death, called red death, is observed in response to PAO1 grown on phosphate-depleted medium in conjunction with physiological stress on the nematodes [10].

**Figure 1:**
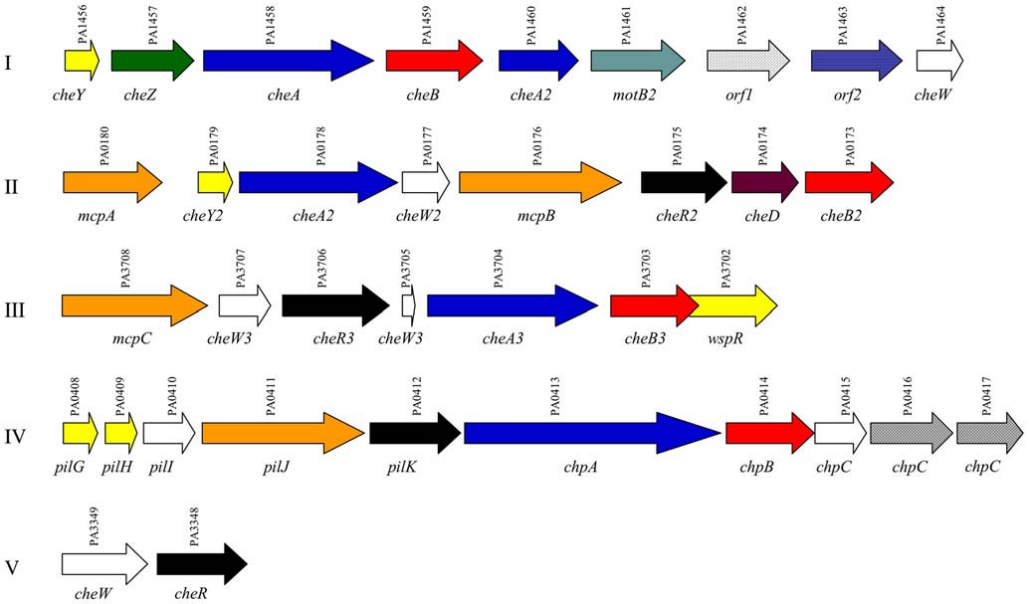
Chemotaxis Clusters in *P. aeruginosa* PAO1. Cluster numbers are indicated to the left. Gene names are indicated below each representative arrow bar and PA numbers (http://pseudomonas.com) are indicated above the arrow bars. Homologues are indicated by shared color.

It is known that the *P. aeruginosa* isolates PA14 and PAO1 show genomic diversity. Strains cultured *in vitro* for years are known to undergo changes in gene expression. Individual genes and even entire genomic islands have been lost from laboratory isolates when compared to pathogenic strains [11],[12],[13]. In this study, we used the *P. aeruginosa* strain TBCF10839 (TB), which was isolated from a Cystic Fibrosis (CF) patient [14]. The TB strain belongs to an abundant clonal complex in the *P. aeruginosa* population [15]. It has a high resistance to detergents [16] and reactive oxygen intermediates [17] and is able to grow within polymorphonuclear leukocytes [18]. We developed a high-throughput killing assay using *C. elegans* as a host, which will be appropriate for a systematic screen of mutant libraries of any *P. aeruginosa* isolate of interest. Increasing throughput in an assay such that an entire genome can readily be scanned in a short time period is an important advance. Our protocol is based on a standard killing assay and makes use of a Biosorter to distribute nematodes into the wells of microtitre plates in a fully automated manner. We screened a TB STM (signature tagged mutagenesis) mutant library of 2,200 non-redundant clones [19],[20] and selected a small group of mutants, significantly attenuated for virulence in *C. elegans*. By testing these mutants in additional phenotypic assays, including adherence to epithelial cells and virulence in a mouse model, we identified a gene, *cheB2*, necessary for *in vivo P. aeruginosa* virulence. The *cheB2* gene belongs to the *P. aeruginosa* chemotaxis *che2* gene cluster (cluster II, *PA0180-PA0173*) (Figure 1) [21]. *P. aeruginosa* has multiple copies of chemotaxis genes arranged in five clusters (Figure 1) [22]. The chemotaxis gene cluster I (*PA1456-PA1464*) and cluster V (*PA3349-PA3348*) have previously been shown to be essential for chemotaxis and flagellar mobility in *P. aeruginosa* [23],[24]. The *wsp* genes contained in cluster III (*PA3708-PA3702*) have been proposed to contribute to bacterial biofilm formation [25], whereas the cluster IV (Pil-Chp system, *PA0408-PA0417*) has been shown to be involved in twitching motility [26],[27]. The *che2* genes from cluster II, and more particularly the *cheB2* gene, were initially identified as required for an optimal chemotactic response in *P. aeruginosa*. The CheB proteins are essential components of the chemotactic response and are responsible for demethylation of glutamate residues in methyl-accepting chemotaxis proteins (MCPs) and also deamidation of glutamine residues to form methyl-accepting glutamates [28]. MCPs sense the chemical stimuli that initiate the chemotactic activity [29]. In *Escherichia coli*, the loss of CheB leads decreased receptor sensitivity, due to an inability to reset the MCP's [30]. This modification leaves the chemotactic pathway in active states causing clockwise rotation of the flagella and continuous tumbling instead of runs and tumbles. It has been reported previously in *Salmonella typhimurium* that a loss of proper chemotaxis control through a *cheB* mutation, leads to a ‘tumbly’ swimming phenotype and a strong reduction in the isolates ability to invade human epithelial HEP2 cells as well as a reduction in infectivity in a mouse ligated-loop model [31].

In our study we further compare the phenotypes of *cheB2* and *cheB1* mutants and propose that the *cheB2* gene has a specific role during infection, which is essential for pathogenesis.

## Results

### High Throughput Assay for Nematode Killing by *P. aeruginosa*

It was reported earlier that relatively high-throughput screening procedures performed on plates could be used to identify virulence factors in various pathogens such as *Staphylococcus aureus* [32], *P. aeruginosa* [8] or *Serratia marcescens* [33]. Here, we developed a high throughput screening method, in which *C. elegans* killing is assessed in a liquid assay and which allows a quick selection of attenuated candidates within large transposon libraries of *P. aeruginosa*. We prepared a bacterial culture medium that both allowed the worms to thrive feeding on the non-pathogenic *E. coli* OP50, and bacterial isolates being tested to have normal (see Materials and Methods). We filled 96 well plates with the medium and inoculated the bacterial strains to be tested. The virulence of each isolate was assessed in a second microtitre plate, into which a specific population of nematodes had been deposited. Evaluation of virulence was based upon the number of live worms recovered after 24 h exposure to bacteria. Under our assay conditions, more than 90% of the worms grown on *E. coli* were still alive after 24 h. In contrast, more than 50% of the worms were killed within 24 h when cultured with any one of three isolates of *P. aeruginosa*, PA14, PAO1 and TB (data not shown). In order to confirm that this assay would allow the recovery of *P. aeruginosa* mutants with an attenuated virulence, we tested previously characterized mutants. We used a *gacA* mutant from the PA14 transposon library (http://ausubellab.mgh.harvard.edu/cgi-bin/pa14/home.cgi) [34], which is affected in a gene encoding a response regulator involved in virulence [8]. *C. elegans* infection using this mutant in the liquid assay resulted in only 40% killing at 24 h, whereas 95% killing was observed with the parental PA14 strain (data not shown).

In order to screen a large number of bacterial isolates more rapidly, the assay was automated using the COPAS (Complex Object Parametric Analyzer and Sorter) Biosort system (Union Biometrica) (http://www.unionbio.com/). The sorter is able to dispatch systematically a fixed number of worms of a precise developmental stage, into each well of a microtitre plate.

### Screening the TB Transposon Library for Mutants Attenuated in Virulence

A signature tagged mutagenesis (STM) library was previously generated in the virulent *P. aeruginosa* CF isolate, TB, using the plasposon vector pTnModOGm [20],[35]. Auxotrophic mutants were removed from the library by pre-screening post-conjugation TB isolates onto minimal media and selecting only clones, which grew normally. This pre-screening eliminated clones that might have exhibited attenuated virulence based upon reduced growth or viability, rather than loss of a specific virulence function. After this initial segregation, 2,200 clones were picked into a 96-well plate and tested with the *C. elegans* high throughput-killing assay. Each mutant was tested at least four times using this assay. The mutants selected were segregated into three groups based upon their level of virulence attenuation as compared to the TB parental strain, which killed 85% of worms under the test conditions. In group 1, we included 187 mutants (9% of total library) which allowed more than 50% worm survival, group 2 between 30% and 50% (536 mutants, 25%), and group 3 below 30% (1450 mutants, 65%), approaching the same level of killing as the parental isolate. Within group 1, the 12 most highly attenuated isolates, classified as group 1A (0.4% of the total library), were further studied. When cultured in the presence of these mutants more than 70% of worms survived, representing a near 5-fold reduction in virulence compared to TB.

### Characterization of Selected Attenuated Mutants

Genomic DNA was recovered from the 12 group 1A isolates. The transposon insertion site was determined through direct genomic DNA sequencing, using primers located upstream from each transposon inverted repeat (see Materials and Methods). The sequences obtained were compared to the PAO1 genome sequence database (http://www.pseudomonas.com) [22] followed by comparison with the general sequence database. The genes identified are shown in Table 1. Two potential regulatory genes were found. The first, *PA2588*, encodes an AraC-type regulatory protein, with 48% similarity to the PAO1 *PA0831* gene product, OruR [36]. The second matched *PA4380*, which encodes a protein with 76% similarity to *Pseudomonas fluorescens* ColS, a histidine kinase sensor from a two-component regulatory system. In *P. fluorescens* ColS has been implicated in root colonization while in *Pseudomonas putida* it has been reported to be involved in regulating TN4652 transposition and heavy metal resistance [37],[38],[39],[40]. Another gene was identified as *PA4554*, which encodes the type 4 fimbrial adhesin PilY1 [41]. We recovered two additional mutants with transposon insertion into genes which have functions related to motility. First, *PA4953* encodes a protein with similarity to the *E. coli* MotB, one of a pair of proteins, which contributes to flagellar rotation [23],[42],[43]. Secondly, we found an insertion in *PA0173* (*cheB2*), one of four *cheB* gene homologues in the *P. aeruginosa* genome sequence. *PA0173* lies within the chemotactic gene cluster II found in the PAO1 genome (Figure 1) [21]. CheB proteins are responsible for removing methyl groups from MCPs [44]. The adaptation of the chemotaxis system in response to changes in attractant binding is dependent on the methylation state of the MCPs. CheB2 has been proposed to be an essential component for an optimal chemotactic response [21]. In the remaining mutants, one contained a transposon insertion in *PA5479*, which encodes a protein similar to GltP from *E. coli*, a glutamate-aspartate carrier protein. Another mutant had an insertion in *PA2585* encoding the UvrABC endonuclease subunit UvrC, required for the excision of damaged DNA. The *uvrC* gene is located downstream from the *gacA* gene encoding a response regulator known to be involved in *P. aeruginosa* virulence. Since, in addition to the insertion in *PA2585*, we isolated a mutant with an insertion in a neighbor gene *PA2588*, it suggests that this cluster of genes might be required for virulence. Another interrupted gene, *PA2478*, encodes a probable thiol∶disulfide interchange protein of the DsbD family. Finally, 4 interrupted genes, *PA0946*, *PA3080*, *PA0260* and *PA2769*, encode proteins of unknown function.

**Table 1:** Identification and characterization of selected mutants.

ORF/STM mutant	Function	Gene name	Slow killing	Cell Adherence	Swimming	Swarming
PA0173/**TB0173s**	probable methylesterase for chemotaxis	*cheB2*	**−**	**−**	**−**	**−**
PA0260/**TB0260s**	hypothetical protein		**−**	**+**	**−**	**+**
PA0946/**TB0946s**	hypothetical protein		**−**	**+**	**+**	**+**
PA2478/**TB2478s**	probable thiol∶disulfide interchange protein	*dsbD*2	**+**	**+**	**+**	**+**
PA2585/**TB2585s**	endonuclease UvrABC subunit C	*uvrC*	**−**	**+**	**+**	**+**
PA2588/**TB2588s**	probable transcriptional regulator		**+**	**−**	**+**	**−**
PA2769/**TB2769s**	hypothetical protein		**+**	**−**	**+**	**+**
PA3080/**TB3080s**	hypothetical protein		**−**	**−**	**+**	**−**
PA4380/**TB4380s**	probable histidine kinase sensor	*colS*	**−**	**−**	**+**	**+**
PA4554/**TB4554s**	type 4 fimbrial biogenesis protein	*pilY*1	**−**	**+/−**	**+**	**+**
PA4953/**TB4953s**	flagellar motor protein	*motB*	**+**	**−**	**−**	**−**
PA5479/**TB5479s**	proton-glutamate symporter	*gltP*	**+**	**−**	**−**	**+**

ORF PA number, function and gene name annotations were obtained using the Pseudomonas genome database version 2 (http://v2.pseudomonas.com/index.jsp).

+ indicates that the strain exhibited a phenotype not significantly different from the wild type TB isolate, whereas − indicates a reduced phenotype. +/− indicates a reduction at the limit of significance.

In order to compare our liquid assay with the established *C. elegans* slow killing assay, we compared the lethality of the TB strain with the previously tested PA14 strain. We observed that both strains kill worms with similar efficiency (data not shown). Each of the 12 group 1A mutants derived from TB was further tested in the slow killing assay. The parental TB isolate is able to kill 50% of exposed nematodes in the slow killing assay within 3 days. Seven of the twelve group 1A isolates showed significant virulence attenuation (Table 1). For example, three group 1A mutants TB0173s (*cheB2*, p<0.001), TB4554s (*pilY1*, p<0.001) and TB4380s (*colS*, p<0.001) each required at least one additional day (4 days) in order to reach 50% killing (data not shown). Survival curves are considered significantly different from the control when p-values are <0.05 (see Materials and Methods).

### Phenotypic Analysis of Selected TB mutants

Several of the clones recovered were mutated in genes putatively associated with adherence and motility, such as *cheB2* (*PA0173*), *motB* (*PA4953*) and *pilY*1 (*PA4554*). It has been shown that adherence is an essential first step in the process of *P. aeruginosa* colonization, allowing the bacteria to persist, expand in numbers and develop a biofilm or establish intimate contact with host cells [45],[46]. We wished to determine if our selected mutants showed a reduction in their capacity to adhere to a biological surface such as the human epithelial cell line 16HBE14o-. In addition to TB4953s (*motB*, p<0.0001) and TB0173s (*cheB2*, p<0.001), that presented strongly reduced adherence, five other clones were significantly affected in their adherence to cells when compared to the parental TB isolate (Figure S1 and Table 1). These mutants were TB2588s (*araC-like*, *PA2588*, p<0.001), TB4380s (*colS-like*, *PA4380*, p<0.001), TB3080s (hypothetical, *PA3080*, p<0.01), TB2769s (hypothetical, *PA2769*, p<0.05) and TB5479s (*gltP-like*, *PA5479*, p<0.05).

**Figure 2:**
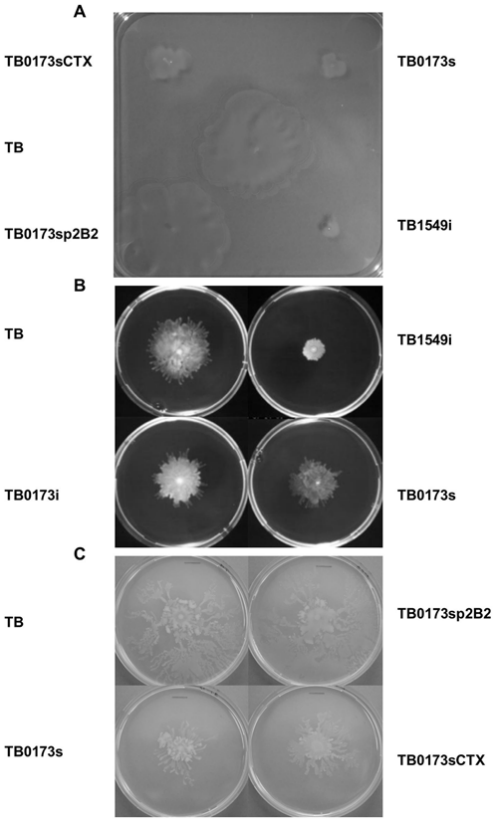
Swimming and Swarming Motility of TB and Isogenic Mutants. (A) Swimming motility. The *cheB2* mutant (TB0173s) shows reduced motility as compared to the parental strain (TB) whereas complementation with CTXp2B2 (TB0173sp2B2) fully restores motility. The non-complemented *cheB2* mutant strain carrying CTX1 (TB0173sCTX) remains poorly motile. The *cheB1* mutant (TB1549i) displays greater motility defect as compared to the *cheB2* mutant. Colony area representing the motility zone was calculated using Macnification software (Orbicule BVBA Heverlee, Belgium). TB; 15,92 cm^2^, TB0173s (*cheB2* mutant); 2,58 cm^2^, TB0173sp2B2 (complemented *cheB2* mutant); 14,25 cm^2^, TB0173CTX (*cheB2* mutant); 1,0 cm^2^, TB1549i (*cheB1* mutant); 0,48 cm^2^. (B) Swarming motility. The *cheB2* mutants (TB0173s and TB0173i) display slightly reduced motility as compared to the parental strain (TB), whereas the *cheB1* mutant (TB1549i) shows a drastic swarming motility defect. (C) Swarming motility of the *cheB2* mutant (TB0173s) is restored to wild-type strain (TB) level upon introduction of the CTXp2B2 (TB0173sp2B2), whereas the *cheB2* mutant strain carrying an empty CTX1 (TB0173sCTX) does not display a similar motility restoration.

Motility is also an important factor for *P. aeruginosa* colonization and spread. We evaluated each of the twelve attenuated mutants for both swarming and swimming motility (data not shown, Table 1 and Figure 2). The TB0173s (*cheB2*) (Figure 2 and Table 1) and TB4953s (*motB*) (Table 1) mutants showed a reduction in swimming and swarming motility, respectively. Furthermore, the mutants TB0260s (hypothetical, *PA0260*) and TB5479s (*gltP-like*) had a significant reduction in swimming motility (Table 1) whereas the mutants TB2588s (*araC-like*) and TB3080s (hypothetical) had a significant reduction in swarming motility (Table 1).

### Characterization of the *cheB2* Mutant

We chose to further characterize the TB0173s mutant (*cheB2*), as this isolate was affected in all phenotypes tested and thus exhibited a general motility and virulence defect (Table 1). To ensure that the *cheB2* gene in the TB strain is located in a cluster similar to the *che2* cluster described for the PAO1 or PA14 strains, we further analyzed the corresponding TB chromosomal region. A series of 8 PCR reactions were performed using oligonucleotides based on the nucleotide sequence obtained from the PA14 genome sequence (Table S1). The 8 PCR products were meant to cover the totality of the *che2* cluster (Figure 1 and Figure S2) starting from the *mcpA* gene to the *PA0171* gene located downstream *cheB2* (*PA0173*). The reactions were performed simultaneously using PA14 or TB genomic DNA as matrix. Each paired reaction set (TB/PA14) resulted in products running at the same migratory rate within the 1.5% agarose gel (data not shown). These results show that there is little difference between the two isolates, TB and PA14, with regards the overall organization of the *che2* chromosomal region.

**Figure 3:**
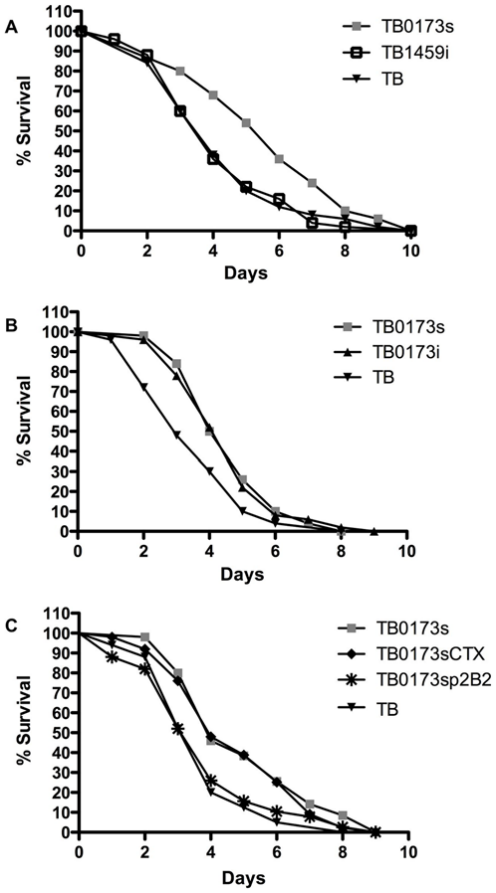
*C. elegans* Slow Killing Assay. (A) *C. elegans* survival assay comparison between the parental strain TB, the original *cheB2* mutant from the STM library (TB0173s) and the reengineered *cheB2* insertion mutant (TB0173i). (B) Comparison between, TB0173s (*cheB2* mutant), TB0173sCTX (*cheB2* mutant harboring CTX1), TB0173sp2B2 (complemented *cheB2* mutant after chromosomal integration of CTXp2B2 carrying the wild type *cheB2* gene) and the parental strain (TB). (C) Comparison between, the parental strain TB, the original *cheB2* mutant (TB0173s) and the engineered *cheB1* mutant (TB1459i). The percent of nematode survival (y axis) is shown with respect to the number of days post-infection (x axis).

To validate that the loss of virulence and other phenotypic changes observed with the TB strain were a result of the transposon disruption of the *cheB2* gene, we generated a new mutant by interrupting the *cheB2* gene (see Materials and Methods). As shown in Figure 3A, the newly engineered *cheB2* mutant, called TB0173i (p<0.005), showed a delayed *C. elegans* killing comparable to the original STM *cheB2* mutant TB0173s (p<0.004). Both *cheB2* mutants, TB0173s and TB0173i, required at least one additional day, as compared to TB, to kill 50% of exposed nematodes. In addition, we engineered a similar *cheB2* mutation in the *P. aeruginosa* PA14 strain, yielding PA140173i (see Materials and Methods), and observed similar virulence attenuation on *C. elegans* (Figure S3). This further confirmed the virulence phenotype of a *cheB2* mutant and indicated that it is not a TB strain-specific trait. Finally, a complementation assay was performed. We reintroduced, in the *cheB2* mutant, the wild-type *cheB2* gene using an integration-proficient vector, mini-CTX1 [47]. This vector contains the ϕCTX attachment site allowing integration of exogenous DNA fragments at the *attB* site within the genome of *P. aeruginosa*. The *cheB2* gene was cloned into the mini-CTX1, yielding CTXp2B2 (see Materials and Methods). This construct was introduced into the *cheB2* mutant, TB0173s, generating the TB0173sp2B2 strain. The empty vector, mini-CTX1, was also introduced into the TB0173s *cheB2* mutant, generating TB0173sCTX. As shown in Figure 3B the virulence was restored in the complemented *cheB2* mutant TB0173sp2B2 (p<0.009), whereas the *cheB2* mutant strain TB0173sCTX (p<0.005) remained fully attenuated.

### A *cheB1* Mutant Is not Attenuated for *C. elegans* Virulence

Several chemotactic gene clusters have been identified in the *P. aeruginosa* genome [21],[23]. The *che1* gene cluster encodes the principle chemotactic device controlling flagellar mobility [23]. We investigated whether the loss of virulence observed in the *cheB2* mutant on *C. elegans* is specific, and results from a loss of chemotaxis under the direction of the *che2* chemotactic cluster, rather than being linked with more global chemotactic or motility deficiency. We thus investigated the fate of a *cheB1* mutant in a similar assay. The *cheB1* mutation was engineered in the TB strain as described in materials and methods, yielding TB1459i. The *cheB1* mutant was used to feed *C. elegans* in the slow killing assay and the survival percentage was compared to those obtained when using the *cheB2* mutant or the parental TB strain (Figure 3C). Interestingly, the *cheB1* mutant showed no significant reduction in *C. elegans* killing when compared to the parental TB strain, with only 36% of the worms alive after 4 days post infection. This is in contrast to the loss of virulence in the original *cheB2* mutant (TB0173s) and the insertional *cheB2* mutant TB0173i.

The mobility of the *cheB1* mutant was also analyzed and both swimming and swarming motility appeared to be affected as shown in Figure 2. The swimming or swarming motility defect is, however, much more severe in the *cheB1* mutant as compared to the *cheB2* mutant. Finally, we used a chemotactic assay using tryptone as chemo-attractant (see Materials and Methods). The parental TB strain was able to move towards the attractant (an increase of slightly more than half a log of bacteria over 45 min) and the TB0173s strain (*cheB2* mutant) was also capable of chemotaxis, even though much reduced as compared to TB (Figure S4). However, the TB1459i strain (*cheB1* mutant) was no longer able to move towards the attractant, and thus showed a defect in this chemotaxis assay (Figure S4).

### The *cheB2* Mutant Shows Reduced Virulence in Mice

**Figure 4:**
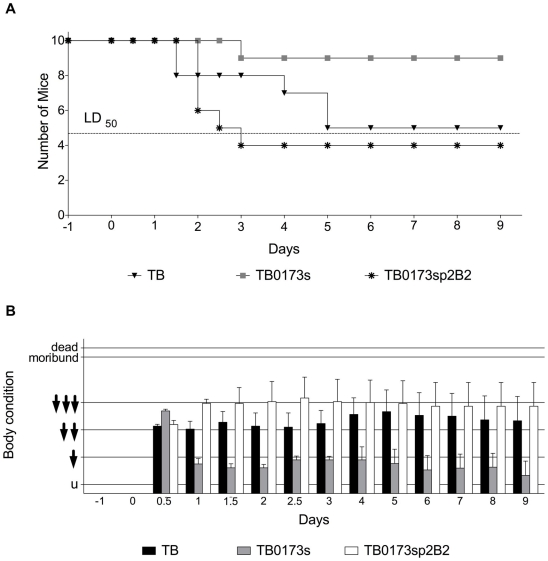
Mouse Lung Infection with *P. aeruginosa* TB and Mutant *cheB2*. Panels A–B show the outcome of a representative single infection experiment with groups of 10 mice each. The impact on virulence of the parental TB, the *cheB2* mutant TB0173s or the complemented *cheB2* mutant TB0173sp2B2 isolate in a mouse lung infection model was tested. (A) The TB parental strain showed a maximal killing rate 5 days post-infection while the *cheB2* mutant strain TB0173s is severely impaired for virulence. The complemented strain, TB0173sp2B2, like the parental TB isolate, is fully virulent and reaches a maximal killing by day 3. Dashed line indicates the LD_50_. (B) Mouse body condition and behavior was evaluated following infection. While the mice infected with the *cheB2* mutant TB0173s, showed a generally untroubled condition throughout the experiment, the mice infected with the parental strain or the complemented *cheB2* mutant strain (TB0173sp2B2) experienced much more severe dysfunction in condition and behavior.

We further tested the virulence of the *cheB2* mutant TB0173s in a mouse model of lung infection, utilizing the view-controlled intratracheal infection protocol [48]. Ten female C3H/HeN mice each were inoculated with 7.5×10^6^ CFU equivalent to the LD_50_ dose of the wild type strain determined in prior experiments (data not shown). Figure 4 displays the representative outcome of one of three independent infection experiments. The *cheB2* mutant (TB0173s) showed a significantly attenuated virulence during respiratory tract infection of mice as compared to the parental strain TB and the complemented strain TB0173sp2B2. Infection with the wild type resulted in 50% dead animals (5 of 10) by day 5 compared to 10% deaths for the TB0173s cheB2 mutant (1 of 10; P = 0.036, Fisher's exact test). Infection with the complemented strain TB0173sp2B2 resulted in 60% dead by day 3 (6 of 10; P = 0,0018, Fisher's exact test) (Figure 4A). We have also evaluated the body condition of the mice during bacterial infection (see Materials and Methods). Surviving mice infected with the *cheB2* mutant showed a significantly less troubled body condition from days 3 to 6 post infection (P<0.01, Mann-Whitney rank test) than surviving mice infected with the wild type strain indicating that the former group recovered earlier than the latter (Figure 4B).

### Pathohistological Analysis of *P. aeruginosa* Infection

**Figure 5:**
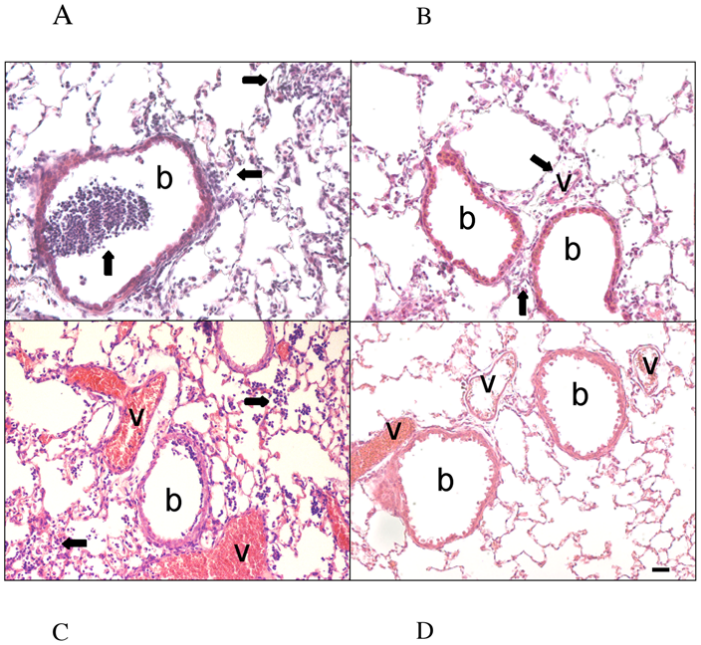
Pathohistological Signs of Inflammation in Murine Lungs. Two days after intratracheal infection with *P. aeruginosa* TB or the *cheB2* mutant TB0173s, mice were sacrificed. Inflammatory infiltrates are marked by arrows; b: bronchus, v: vessel. (A) TB shows a strong purulent inflammation with intra- and peribronchiolar infiltrates of leucocytes. (B) Slight peribronchiolar and perivascular inflammation is seen with the *cheB2* mutant TB0173s. (C) The complemented strain TB0173sp2B2, like the wild type TB isolate, shows a strong purulent inflammation with intra- and peribronchiolar infiltrates of leucocytes. (D) The vehicle control was instilled with 30 µl PBS. Magnification×200.

To further evaluate the change in virulence between the parental TB isolate and the TB0173s *cheB2* mutant, mice were sacrificed 2 days after bacterial challenge and the lungs were formalin fixed and paraffin embedded. Lungs from infected mice challenged with the TB strain as well as the complemented TB0173sp2B2 strain had a pronounced polymorphonuclear neutrophil (PMN) infiltration as well as strong peribronchiolar and perivascular inflammation (Figure 5A). In contrast, the TB0173s *cheB2* mutant had caused markedly reduced inflammation and very little PMN infiltration (Figure 5B).

## Discussion

*C. elegans* is a convenient model for studying bacterial virulence mechanisms [7],[49] and it was shown that virulence factors important in the killing of *C. elegans* are relevant for virulence in mammalian hosts [50],[51]. Growth conditions and host-pathogen interactions affect the physiology of *P. aeruginosa* via complex regulatory networks, which in turn control an arsenal of virulence factors [52],[53],[54],[55]. We developed a liquid-based high throughput *C. elegans* killing assay to speed the screening process of bacterial libraries and to perform multiple screens on virulent clinical isolates. This method was automated using the COPAS Biosort worm sorting system, which greatly reduced the time required to perform the assay. Under slow killing conditions (Figure 3) the TB strain requires approximately three days to kill 50% of a given worm population, and up to 8 days to kill all the worms in a population of 50 nematodes. The liquid killing assay by contrast, is read after overnight feeding of the nematodes with the bacterial strains. It is worth noting that a mutant affected in the *gacA* gene, previously identified in the slow killing assay, appeared to be attenuated in our liquid-based killing assay.

We used our killing assay to screen a STM library of the *P. aeruginosa* CF isolate TB [20]. We identified several factors, not yet associated with virulence in *P. aeruginosa*. Several of the genes identified relate to regulatory, motility or unknown functions. We tested additional virulence and motility related phenotypes including *C. elegans* slow killing, swarming and swimming motility, or adherence to human cells (Table 1). Most of our selected mutants were impaired in at least one of the additional tested phenotypes.

One of the identified mutants showed reductions in all aspects of virulence we tested. This mutant, named TB0173s, has a transposon insertion in *PA0173*, *cheB2*, one of four *cheB* genes found in *P. aeruginosa*. CheB protein functions involve interactions with MCPs. As many as 26 MCPs have been identified in *P. aeruginosa* [56], and it has been proposed that the CheB2 protein functions at least with McpB [57].

In the high throughput *C. elegans* liquid-based killing assay and the slow killing assay, the *cheB2* mutant showed significant virulence attenuation. The *cheB2* mutant was also found to have reduced adhesive capabilities to bronchial epithelial cells (Figure S1). Finally, the reductions seen in the motility assays, swimming and swarming, are in agreement with previously reported results revealing a partial loss of motility capabilities [21]. Most importantly in a mouse lung model of infection, the *cheB2* mutant was highly attenuated and failed to induce strong inflammatory response in the infected mice lungs. Interestingly, we observed that a mutation in the *cheB1* gene yields different phenotypes compared to the *cheB2* mutation. The *cheB1* mutant virulence is not attenuated in the slow killing assay. Moreover, it shows more severe motility defect than the *cheB2* mutant. Finally, in chemotaxis assays using tryptone as chemo-attractant, we observed that, while the *cheB1* mutant was strongly affected, the *cheB2* mutant had a mild phenotype. These observations suggest that the attenuation in virulence of the *cheB2* mutant is specific and not due to a global effect on motility or chemotaxis.

As cluster I of *che* genes plays the dominant role in *P. aeruginosa* chemotaxis and flagellar mobility, cluster II genes may be induced under very specific conditions and function in fine-tuning the bacterial response to conditions encountered within a host. Schuster and collaborators have reported that the cluster II genes are induced during stationary phase, are regulated by quorum sensing and that RpoS, the stationary phase RNA polymerase sigma factor, plays a role in controlling *cheB2* gene expression [58]. Moreover, Burrowes and collaborators reported that RsmA exerted control over *cheB2* with a 10-fold reduction in expression of *cheB2* in an *rsmA* mutant [59]. RsmA works in conjunction with small non-coding RNA to regulate the expression of multiple virulence genes, including the quorum sensing *lasI* and *rhlI* genes [60]. Finally, using fluorescent protein-tagged CheY and CheA, it was shown that the Che1 proteins (cluster I) localized to the flagellated pole throughout growth [61]. While the Che1 proteins are still found as bacterial cells entered stationary phase, a patch of Che2 proteins begins to co-localize with the Che1 proteins at that stage. This might indicate that during stationary phase, the chemotactic response will be different than the one observed with exponentially growing cells and the function of the Che2 cluster, even though currently unknown, might be to respond to particular stimuli encountered at that stage.

It has been shown that a *cheW* mutant in *Helicobacter pylori* is unable to establish a normal long-term infection in mice, remaining only in one portion of the stomach. The bacterial pathogen may lose its ability to perceive its niche due to the absence of chemotaxis [62]. It is a possibility that within a host, the cluster II *che* genes of *P. aeruginosa* might play a similar role in sensing particular conditions during infection.

As previously mentioned, even though the *cheB2* mutant is impaired in motility, it is likely that the *cheB2* defect in virulence is not linked to this phenotype but to additional traits of virulence and attachment that have not yet been determined. We have shown that the *cheB1* mutant is affected in motility but still is virulent. Furthermore, in our study, we isolated a second mutant with reduced motility TB4953s (*motB* mutant) (Table 1), and while this isolate was attenuated in the high throughput assay, it did not show a reduction in killing in the slow assay (data not shown), further suggesting that the loss of virulence seen in the *cheB2* mutant strain (TB0173s) is not solely due to a reduction or change in motility.

While some bacteria require host-derived factors for their growth and are difficult to grow *in vitro*, others, such as *S. marcescens* during its infection of *C. elegans* [33], grow at a much lower rate in the host compared to *in vitro*. Our observations support the hypothesis that the *che2* gene cluster plays a major role during host colonization. For example, we may hypothesize that within the gut of the nematode, or the lungs of the mice, bacteria might reach high cell density and develop as biofilm. This could be a condition to set the function of the Che2 proteins and allow non-growing cells to sense and respond to the environment in a different manner as compared to fast-growing cells. While the idea that the Che2 system might be a specialized chemotaxis system required during host infection is appealing, further experiments are required to understand the chain of command, which switches on the Che2 system and directs the physiological response of *P. aeruginosa* to Che2 sensing.

## Materials and Methods

### Bacterial strains and growth conditions

Bacterial strains and plasmids used are listed in Table 2. The *P. aeruginosa* TB STM transposon library was constructed with the plasposon pTnModOGm [35] carrying variable signature tags as described previously [20]. Plasmids were introduced into *P. aeruginosa* strains by mating using pRK2013 as a helper or through electroporation. The transformants were selected on *Pseudomonas* isolation agar containing antibiotics. Plasmids were maintained by adding ampicillin for *E. coli* (50 µg/ml) and carbenicillin, tetracycline and gentamycin for *P. aeruginosa* (300, 200 and 10 µg/ml, respectively).

**Table 2:** Strains and plasmids used in this study.

Strains/Plasmids	Relevant characteristics^*^	Reference/origin
***Escherichia coli***		
TG1	*supE* (*lac-proAB*)*thi hsdR 5*(F′ *traD36 proA*+*B*+*lacI* q*ZM15*)	Lab. collection
TOP10F′	F′ [ *lacI* qTn *10* (Tet r)] *mrcA* (*mrr-hsdRMS-mcrBC*) 80 *lacZ M15 lacX74 recA1 araD139* (*ara-leu*)*7697 galU galK rpsL* (Str r) *endA1 nupG*	Invitrogen
S17 λpir	*thi pro hsdR hsdM*+*recA* RP4-2-Tc::Mu-Km::Tn λ*pir*	Lab. collection
OP50	Uracil auxotroph *E. coli* B	CGC
***Pseudomonas aeruginosa***		
PA14	Wild type	Lab. collection
TBCF10839 (TB)	CF airway wild type, serotype 4	Lab. collection
TB5479s	Tn mutant from the TB STM library, *gltP*, Gm^r^	[19],[20]
TB4953s	Tn mutant from the TB STM library, *motB*, Gm^r^	[19],[20]
TB4554s	Tn mutant from the TB STM library, *pilY1*, Gm^r^	[19],[20]
TB4380s	Tn mutant from the TB STM library, *colS*, Gm^r^	[19],[20]
TB3080s	Tn mutant from the TB STM library, *PA3080*, Gm^r^	[19],[20]
TB2769s	Tn mutant from the TB STM library, *PA2769*, Gm^r^	[19],[20]
TB2588s	Tn mutant from the TB STM library, *PA2588*, Gm^r^	[19],[20]
TB2585s	Tn mutant from the TB STM library, *uvrC*, Gm^r^	[19],[20]
TB2478s	Tn mutant from the TB STM library, *dsbD2*, Gm^r^	[19],[20]
TB0946s	Tn mutant from the TB STM library, *PA0946*, Gm^r^	[19],[20]
TB0260s	Tn mutant from the TB STM library, *PA0260*, Gm^r^	[19],[20]
TB0173s	Tn mutant from the TB STM library, *cheB2*, Gm^r^	[19],[20]
TB0173i	*cheB2* chromosomal insertion mutant derived from TBCF10839. Insertion of PCR2.1 with internal *cheB2* DNA fragment of 667 bp, Cb^r^	This study
TB1459i	*cheB1* chromosomal insertion mutant derived from TBCF10839. Insertion of PCR2.1 with internal *cheB1* DNA fragment of 613 bp, Cb^r^	This study
TB0173sCTX	TB0173s harboring CTX1 on the chromosome, Tc^r^	This study
TB0173sp2B2	TB0173s harboring CTXp2B2 on the chromosome. CTXp2B2 encodes *cheB2* under control of the *mcpA* promoter, Tc^r^	This study
PA140173i	*cheB2* chromosomal insertion mutant derived from PA14. Insertion of PCR2.1 with internal *cheB2* DNA fragment of 667 bp, Cb^r^	This study
**Plasmids**		
pCR2.1	Ap^r^, ColE1 f1 *ori*	Invitrogen
pCR0173	pCR2.1 vector with *cheB2* DNA fragment of 667 bp	This study
pCR1459	pCR2.1 vector with *cheB1* DNA fragment of 612 bp	This study
Mini-CTX1	Tc^r^, *oriT*, modified CTX *int* gene, CTX attachment site	[47]
Mini-CTXp2B2	Tc^r^, Mini-CTX1 with *cheB2* under control of the *mcpA* promoter region	This study

Ap^r^, Ampicillin resistance; Cb^r^, Carbenicillin resistance; Gm^r^, Gentamycin resistance; Tc^r^, Tetracycline resistance.

### Nematode culture

Nematode strains were provided by the *Caenorhabditis* Genetics Center, which is funded by the National Institutes of Health National Center for Research Resources. *C. elegans* strains, wild type N2 (Bristol) and *fer-15*-(*b26*) were cultured as described previously [63]. Eggs were isolated by hypochlorite treatment of gravid adults. *fer-15* eggs were hatched on NGM at 25°C to obtain synchronized sterile worms for use in the slow killing assays. N2 worms were used for all other killing assays.

### Liquid worm assay

Individual clones from the *P. aeruginosa* STM TB library were grown in 96 well plates in liquid assay media (4.0 g NaCl, 2.5 g peptone, 5.0 g tryptone, 2.5 g yeast extract, dH_2_O to 1 liter, 1 ml cholesterol (5 mg/ml stock in ethanol) and 7.5 ml glycerol). Each well was filled with 100 µl of media, inoculated with an individual bacterial clone and incubated 24 h at 37°C. The assay was semi-automated using the Copas Biosort (Union Biometrica). This machine is able to sort worms based on extinction (optical density) and time of flight (length) [64]. Using these two parameters we sorted a population of fifty L4 stage worms into the wells of a new 96-well plate. Then 50 µl of the individual *P. aeruginosa* mutant cultures was transferred to each worm-containing well. Plates were incubated at 25°C overnight with moderate shaking and each well was examined at 24 h with a microscope for surviving worms.

### *C. elegans* slow killing assay

The slow killing assay was performed as described previously [33]. Each independent assay consisted of three replicates. *E. coli* was used as a control. L4 stage *C. elegans* were picked onto plates containing overnight growth of each bacterial strain, and on a daily basis worms were evaluated for viability. Animal survival was plotted using the PRISM 5.00 computer program. Survival curves are considered significantly different from the control when P-values are <0.05. Prism calculates survival fractions using the product limit (Kaplan-Meier) method. Prism compares survival curves by two methods: the log-rank test (also called the Mantel-Cox test) and the Gehan-Breslow-Wilcoxon test.

### Determination of motility

The media used for the swimming assay was tryptone broth (10 g/l tryptone and 5 g/l NaCl) that contained 0.3% (wt/vol) agarose. Swim plates were inoculated with freshly streaked bacteria from an LB agar plate with a sterile toothpick. The plates were placed in a humidified box and incubated at 37°C for 24 h. Swarm agar was based on M9 minimal medium without NH_4_Cl, supplemented with MgSO_4_ (1 mM), glucose (0.2%), and Casamino Acids (0.5%), and solidified with agar (0.5%). Bacteria were spot inoculated on the plates as aliquots taken directly from overnight cultures. Plates were incubated for 16 h at 37°C and incubated an additional 32 h at room temperature [65],[66].

### Adherence assays

16HBE14o- human bronchial epithelial cells were grown in Minimal Essential Media (MEM) supplemented with Fetal Calf Serum (FCS), L-glutamine antibiotics and fungisone. Before infection they were seeded in a 24 well plate with a glass coverslip in each well and incubated overnight at 37°C. At the start of the infection, the MEM was replaced with RPMI 1640 media with 25 mM HEPES without phenol red or FCS for 4 h. They were then infected at an MOI of 40 with one of the bacterial isolates for 1 h at 37°C with appropriate antibiotics. After 1 h non-adherent bacteria were removed from each well of the test plate by repeated washings with phosphate buffered saline (PBS). Each well was fixed using 4% paraformaldehyde and the coverslips mounted for observation. The number of crystal violet stained bacteria adhering to cells was determined by visual evaluation using a Leica microscope. At least 30 cells were examined for each isolate. Statistical analysis was performed using GraphPad 4 software, using a non-parametric t- test.

### Chemotaxis assays

Chemotaxis assays were performed following a modified version of a previously described assay [67]. Bacterial cultures were grown with shaking overnight in LB at 37°C and subcultured into mineral salts media (MSM) [68] supplemented with 0.4% succinate and 7.6 mM ammonium sulfate. The subcultures were incubated for three hours at 37°C to an OD_600_ of approximately 1. The cultures were centrifuged and washed twice with Bushnell-Haas media (BHB) and then tested for chemotaxis using BHB and BHB supplemented with 0.1% tryptone as a chemo-attractant. A 1-ml tuberculin syringe with a disposable 23-gauge needle was filled with 100 µl of (BH) mineral salts medium (Difco) containing 0.1% tryptone as a chemoattractant. A 100 µl sample of the washed bacterial suspension was drawn into a 200 µl pipette filter tip. The syringe was then linked and tightly fit into the tip with 3 mm of the needle inserted into the cell suspension. Duplicate apparatus were incubated at 37°C for 45 min, and the content of the syringe was then diluted in 25 mM PBS and plated onto TSA plates for total CFU.

### DNA manipulations

Cloning, restriction enzyme analysis, and transformation of *E. coli* were performed essentially as described by Sambrook *et al.* [69]. Plasmid DNA was isolated with the QIAprep Spin Miniprep kit, and DNA fragments were purified from agarose gels by using the QIAquick gel extraction kit. Total chromosomal DNA from *P. aeruginosa* was purified with the BioRad AquaPure genomic DNA kit. Direct genomic sequencing was performed by MilleGen Biotechnologies. Primers used for sequencing were IR1f and IR1r (Table 3). Sequences obtained were subjected to BLAST analysis against the PAO1 genome database (http://www.pseudomonas.com) and general databases.

**Table 3:** Oligonucleotides used in this study.

Oligonucleotide name	Sequence#	Position*
IR1f	5′-GCTGCGTTCGGTCAAGGTTC-3′	130 bp downstream from the IR upstream Gm^r^ in pTnMod-OGm
IR1r	5′-CATTTTTTGTGATGCTCGTCAGGG-3′	100 bp upstream of the IR upstream *oriR* in pTnMod-OGm
CheIN1	5′-GACCTGATCAAGCAGCACGC-3′	120 bp downstream from the *cheB2* ATG
CheIN2	5′-CGAACATCACGTCCACTGCC-3′	260 bp upstream from the *cheB2* stop codon
ChebUP	5′-AGCTGAAGAACGACACCGTGC-3′	100 bp upstream of the *cheB2* ATG
ChebDW	5′-TGCGGGGGGGCGAAAAAGC-3′	24 bp downstream the *cheB2* stop codon
Che1INU	5′-GCTGGCCCTGAGACCCGA-3′	131 bp downstream from the *cheB1* ATG
Che1INR	5′-GATGTCGCCGTCCTCTGCT-3′	363 bp upstream from the *cheB1* stop codon
Che1UP	5′-AAGCCACTCGGCAAGATGTTG-3′	154 bp upstream of the *cheB1* ATG
Che1DW	5′-GCGACGAACGCCAGGATG-3′	115 bp downstream the *cheB1* stop codon
B2CTX1	5′-CGT*AAGCTT*GCAGTACGCCTATGCCTACC-3′	393 bp upstream of the *mcpA* (*PA0180*) ATG
B2CTX2	5′-CGT*GAATTC*CAGCTTCAGCAGCAGGGGC-3′	35 bp downstream of the *mcpA* ATG
B2CTX3	5′-TAAC*GAATTC*TCCTGGTGAAGTCCCTGGTGG-3′	130 bp upstream of the *cheB2* ATG
B2CTX4	5′-TAAC*AAGCTT*ACACAATGATGGCGAGCAGGC-3′	100 bp downstream of the *cheB2* stop codon

*: Gm^r^: Gentamycin resistance cassette.

# restriction sites within oligonucleotides are with italics.

### Comparison of the *che2* genomic regions of the TB and PA14 strains

PCR analyses were performed with eight sets of oligonucleotides covering the *che2* cluster-containing region in the PA14 genome (Table S1). These oligonucleotides were used to amplify similar regions within the PA14 and TB genomes. The PA14 and TB genomic DNAs were purified with the BioRad AquaPure genomic DNA kit. PCR reaction products were separated on a 1.5% agarose gel.

### Construction of the *P. aeruginosa cheB2* and *cheB1* mutants

An internal DNA fragment of *cheB*2 was PCR amplified using the primer pair CheIN1 and CheIN2 (Table 3). The resulting 667 bp PCR product was cloned into pCR2.1 yielding pCR0173. The construct was used as a suicide plasmid and introduced into *P. aeruginosa* TB and PA14 strains by electroporation. One selected clone for each strain, TB0173i and PA140173i, respectively, was confirmed for the chromosomal integration event into the *cheB2* gene by PCR using the primer pair ChebUP and ChebDW (Table 3). An internal DNA fragment of *cheB*1 was PCR amplified using the primer pair Che1INU and Che1INR (Table 3). The resulting 613 bp product was cloned into pCR2.1 yielding pCR1459. The construct was used as a suicide plasmid and introduced into *P. aeruginosa* TB strain by electroporation. One selected clone (TB1459i) was confirmed for the chromosomal integration event into the *cheB1* gene by PCR using the primer pair Che1UP and Che1DW (Table 3).

### Complementation of *cheB*2 mutant

The *cheB2* gene along with the putative promoter region for the *che2* chemotaxis cluster located upstream of the *mcpA* gene (*PA0180*) were cloned into the mini-CTX1 vector [64], yielding mini-CTXp2B2. These DNA fragments were PCR amplified using B2CTX1, harboring a *Hin*dIII site, and B2CTX2, harboring an *Eco*R1 site, to amplify a 463 bp-long *mcpA* upstream region, or B2CTX3, harboring an *Eco*R1 site, and B2CTX4, harboring a *Hin*dIII site, to amplify the 1300 bp-long *cheB2*-containing coding region. These two fragments were cloned in the same orientation, with the promoter region preceding the *cheB2* gene. The cloning was done in two steps. Firstly, the promoter region was cloned into the CTX1 using *Eco*R1 and *Hin*dIII. Secondly, this construct was cut using *Eco*R1 and *Bam*H1, the *cheB2*-containing coding region was cut with the same restriction enzymes, taking advantage of a *Bam*H1 site following the coding region by 4 bp. The vector and insert were ligated together generating CTXp2B2. This vector was mated into the *cheB2* mutant TB0173s generating TB0173sp2B2. The empty CTX1 vector was also mated into the *cheB2* mutant TB0173s generating TB0173sCTX. The recombinant clones containing the mini-CTX inserted at the *attB* locus on the *P. aeruginosa* genome were selected on tetracycline-containing PIA plates.

### Murine respiratory tract infection

Prior to animal experiments, bacteria were grown in LB at 37°C with shaking until stationary phase growth was reached. Bacteria were pelleted by centrifugation (5000 g, 10 min), washed twice with PBS. The intended number of CFU was extrapolated from a standard growth curve and appropriate dilutions with sterile PBS were made to prepare the inoculum for the mice. Ten to twelve week old female mice of the inbred strain C3H/HeN were inoculated via view controlled intratracheal instillation with a bacterial suspension containing 7.5×10^6^ CFU [48]. This noninvasive application technique via catheter allows controlled delivery of bacteria to the lungs. During the experiments mice were maintained in micro-isolator cages with filter top lids at 21±2°C, 50±5% humidity and 12 h light/dark cycle. They were fed with autoclaved acidulated water and standard mouse food (SSniff).

To characterize the course of the bacterial infection the weight and rectal temperature of the mice were measured daily and their body condition was determined using a self-developed score. For this score mice behavior was observed and evaluated for the parameters: vocalization, piloerection, attitude, locomotion, breathing, curiosity, nasal secretion, grooming and dehydration. Dysfunctions in single behavioral parameters were assessed by zero, one or two points, respectively. The body condition of the mice was determined by adding the points resulting in the following score: untroubled 0–1 (u); slightly troubled 2–4 (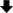); moderately troubled 5–7 (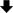 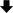); profoundly troubled 8–10 (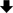 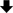 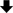); moribund≥11; death≥16. The outcome of the infection experiments was assessed for significant differences between the inoculation of wild type strain and mutant by Fisher's exact test (survival) and Mann-Whitney tests (body condition score).

For pathohistological signs of inflammation, lungs were fixed in 4% formalin and embedded in paraffin. Tissue sections (5-µm) were stained by haematoxylin and eosin and evaluated using a Zeiss light microscope.

## Supporting Information

Figure S1 Adherence capabilities of TB and selected isogenic mutants on epithelial cells. Examination of the binding capacity to 16HBE14o- human airway epithelial cells of the parental TB isolate and mutant clones. Cells were infected at approximately ten to one for one hour with each isolate. Numbers of bacteria per cell were visually quantified by counting numbers of adherent bacteria on at least 30 cells per isolate. (A) Results obtained for each strain were compared to TB strain in unpaired t tests using Graph Pad Prism 4 software. The * indicates the level of significance, ns indicates : not significant. Data shown are mean {plus minus} standard error from three experiments. (B) representative images showing the typical binding of the TB strain and the *cheB2* mutant TB0173s to airway epithelial cells.
(3.00 MB TIF)

Figure S2 Comparison of chemotaxis cluster 2 in *P. aeruginosa* PA14 and TB by PCR analysis. (A) Gene names and PA numbers within cluster 2 are indicated below each representative arrow bar. Oligonucleotide identity is indicated above the cluster, whereas the line indicates their position within the cluster. (B) Bar diagram indicating the overlap between the eight oligonucleotide pairs used.
(3.00 MB TIF)

Figure S3 *C. elegans* slow killing assay. *C. elegans* survival assay comparison between the parental strain PA14 and the *cheB2* insertion mutant (PA140173i). The percent of nematode survival (y axis) is shown with respect to the number of days post-infection (x axis).
(3.00 MB TIF)

Figure S4 Chemotaxis assays. Bacterial cultures were grown in LB at 37°C and subcultured into mineral salts media (MSM) supplemented with succinate and ammonium sulfate. The subcultures were grown to an OD_600_ of approximately 1. The cultures were centrifuged, washed with Bushnell-Haas media (BHB) and tested for chemotaxis using BHB and BHB supplemented with 0.1% tryptone as a chemo-attractant where indicated as 〈〈trypt〉〉. TB is the parental strain, TB1459i is the *cheB1* mutant, and TB0173s is the *cheB2* mutant. Data are mean±standard error of triplicate cultures from three experiments.
(3.00 MB TIF)

Table S1 Oligonucleotides used for *che2* region mapping.
(0.04 MB DOC)
