# Supplementary figures and images for: Caenorhabditis elegans Semi-Automated Liquid Screen Reveals a Specialized Role for the Chemotaxis Gene cheB2 in Pseudomonas aeruginosa Virulence
Source: PLoS Pathog. 2009 Aug 7;5(8):e1000540. doi: 10.1371/journal.ppat.1000540 (PMC2714965; doi:10.1371/journal.ppat.1000540)

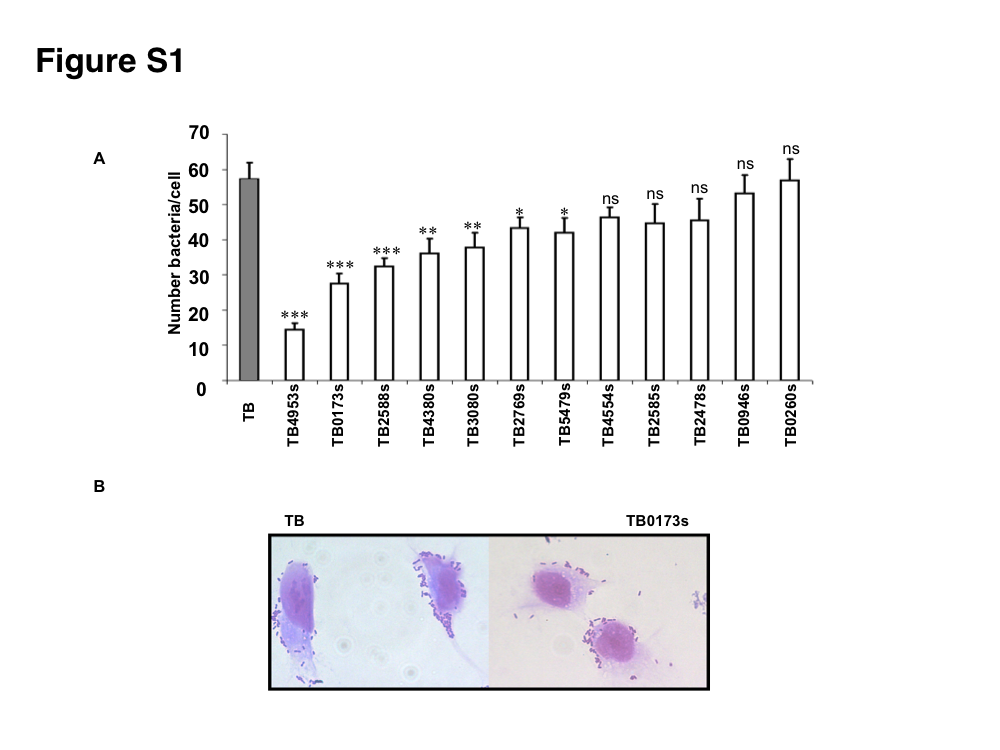

Supplement: Figure S1 — Adherence capabilities of TB and selected isogenic mutants on epithelial cells. Examination of the binding capacity to 16HBE14o- human airway epithelial cells of the parental TB isolate and mutant clones. Cells were infected at approximately ten to one for one hour with each isolate. Numbers of bacteria per cell were visually quantified by counting numbers of adherent bacteria on at least 30 cells per isolate. (A) Results obtained for each strain were compared to TB strain in unpaired t tests using Graph Pad Prism 4 software. The * indicates the level of significance, ns indicates : not significant. Data shown are mean {plus minus} standard error from three experiments. (B) representative images showing the typical binding of the TB strain and the cheB2 mutant TB0173s to airway epithelial cells. (3.00 MB TIF) [file ppat.1000540.s001.tif]

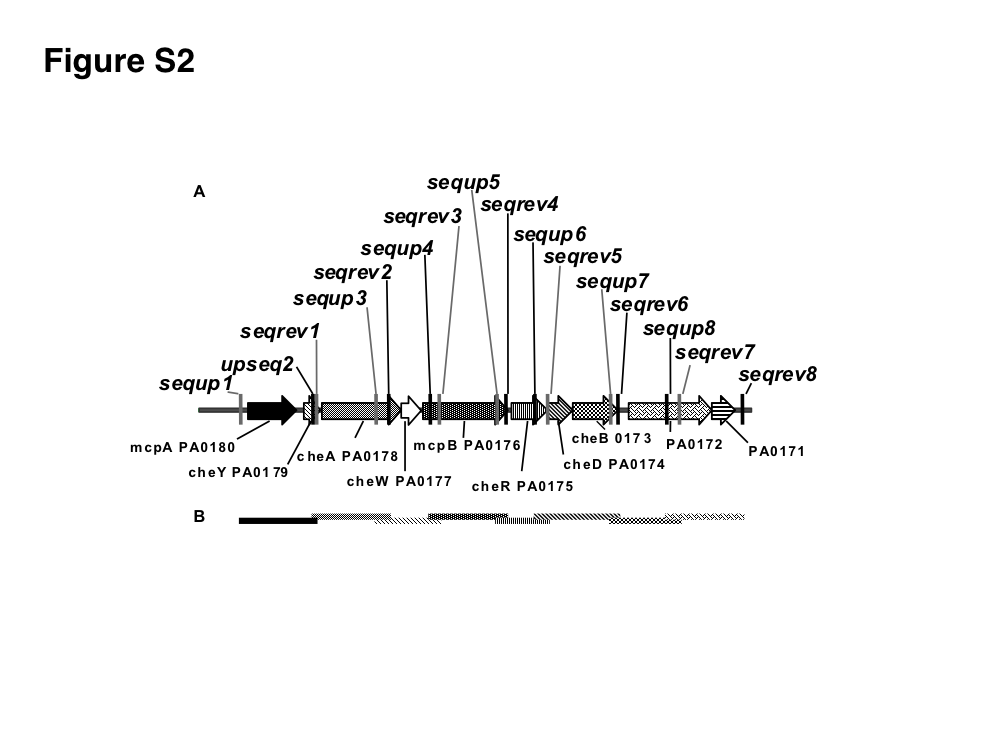

Supplement: Figure S2 — Comparison of chemotaxis cluster 2 in P. aeruginosa PA14 and TB by PCR analysis. (A) Gene names and PA numbers within cluster 2 are indicated below each representative arrow bar. Oligonucleotide identity is indicated above the cluster, whereas the line indicates their position within the cluster. (B) Bar diagram indicating the overlap between the eight oligonucleotide pairs used. (3.00 MB TIF) [file ppat.1000540.s002.tif]

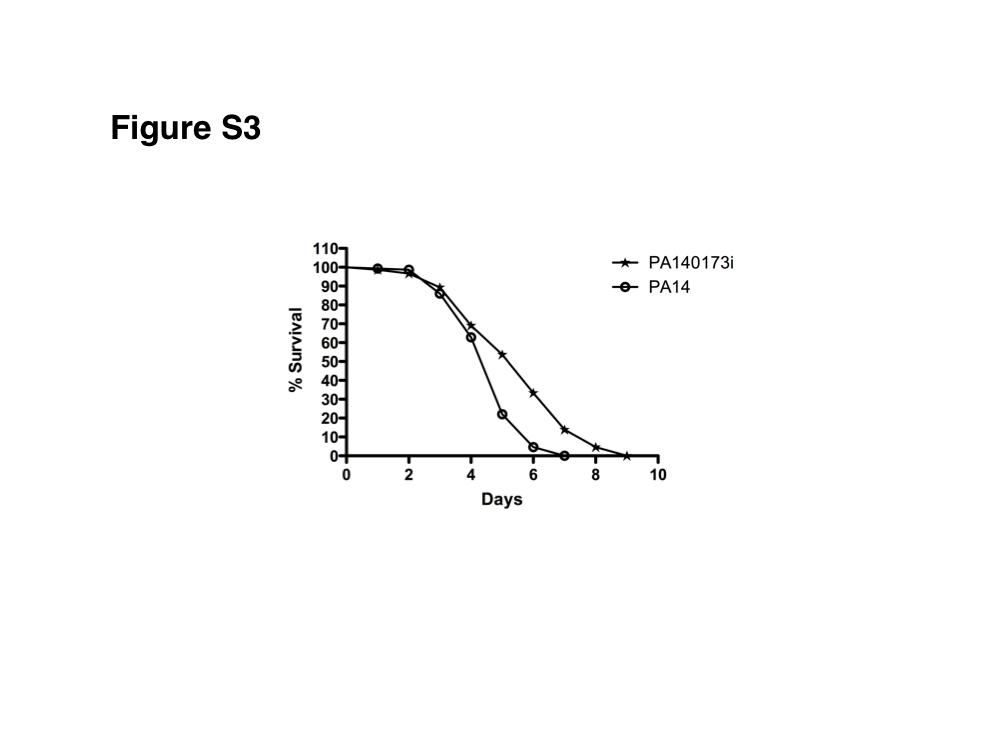

Supplement: Figure S3 — C. elegans slow killing assay. C. elegans survival assay comparison between the parental strain PA14 and the cheB2 insertion mutant (PA140173i). The percent of nematode survival (y axis) is shown with respect to the number of days post-infection (x axis). (3.00 MB TIF) [file ppat.1000540.s003.tif]

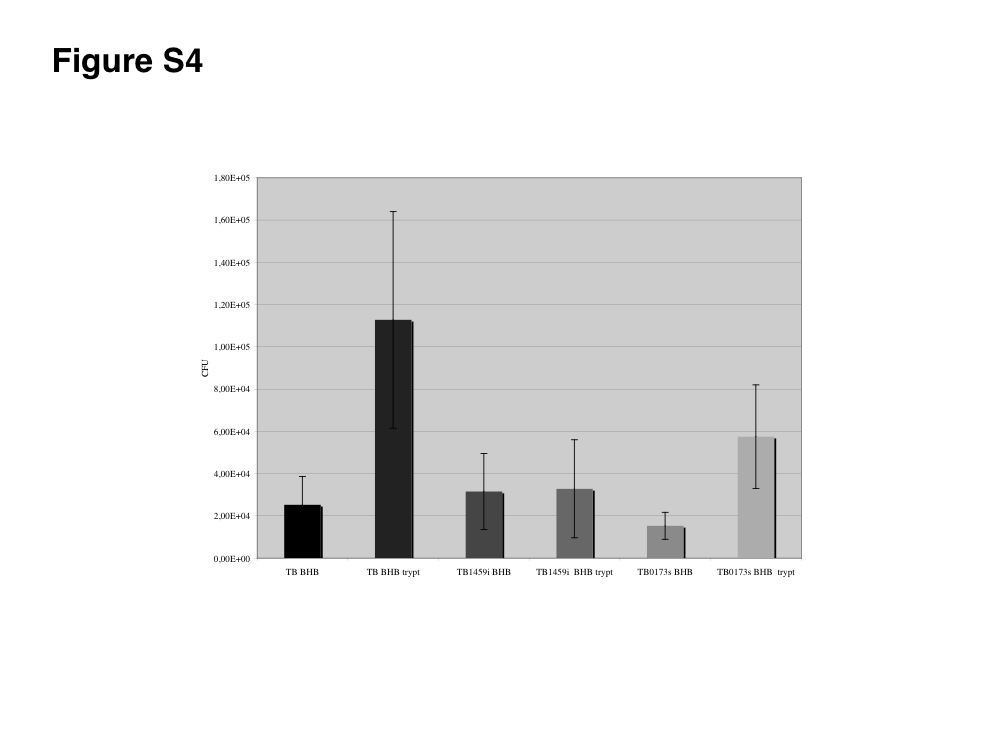

Supplement: Figure S4 — Chemotaxis assays. Bacterial cultures were grown in LB at 37°C and subcultured into mineral salts media (MSM) supplemented with succinate and ammonium sulfate. The subcultures were grown to an OD600 of approximately 1. The cultures were centrifuged, washed with Bushnell-Haas media (BHB) and tested for chemotaxis using BHB and BHB supplemented with 0.1% tryptone as a chemo-attractant where indicated as 〈〈trypt〉〉. TB is the parental strain, TB1459i is the cheB1 mutant, and TB0173s is the cheB2 mutant. Data are mean±standard error of triplicate cultures from three experiments. (3.00 MB TIF) [file ppat.1000540.s004.tif]
