# Supplementary material for: Caenorhabditis elegans Semi-Automated Liquid Screen Reveals a Specialized Role for the Chemotaxis Gene cheB2 in Pseudomonas aeruginosa Virulence
Source: PLoS Pathog. 2009 Aug 7;5(8):e1000540. doi: 10.1371/journal.ppat.1000540 (PMC2714965; doi:10.1371/journal.ppat.1000540)
Supplement: Table S1 — Oligonucleotides used for che2 region mapping. (0.04 MB DOC) [file ppat.1000540.s005.doc]

| **Table S1. Oligonucleotides used for *che2* region mapping** | | |
| --- | --- | --- |
| Oligonucleotide name | Sequence | Position^*^ |
| sequp1 | 5’-tttgacattaccgattgggactctag-3’ | 204811 204786 |
| seqrev1 | 5’-aatcagggcaggaccttgcg-3’ | 202952 202971 |
| sequp2 | 5’-ccgatcctcatgctgaccacc-3’ | 203088 203068 |
| seqrev2 | 5’-cgcaacagctcgtgcaggc-3’ | 201266 201284 |
| sequp3 | 5’-tcggcatggacgtggtgaag-3’ | 201557 201538 |
| seqrev3 | 5’-gatggcttcggtgatctgcg-3’ | 200056 200075 |
| sequp4 | 5’-tggaggcggggctgg-3’ | 200297 200283 |
| seqrev4 | 5’-ggcgtggtctcagaactcttcc-3’ | 198451 198472 |
| sequp5 | 5’-acgacatgacccagcagaacg-3’ | 198707 198687 |
| seqrev5 | 5’-cgaagtacggatcgtagtagcg-3’ | 197485 197506 |
| sequp6 | 5’-aactgcggcaactggtggag-3’ | 197801 197782 |
| seqrev6 | 5’-aggttgcggggggcg-3’ | 195822 195836 |
| sequp7 | 5’-cgaagcggtggaactggg-3’ | 195976 195959 |
| seqrev7 | 5’-aggctggcgtagtcgaggc-3’ | 194335 194353 |
| sequp8 | 5’-acgttcctgctgttgctggtg-3’ | 194654 194634 |
| seqrev8 | 5’-atcgaactggtgttcaggtacagc-3’ | 192828 192851 |

^*^The numbers indicate the position of the oligonucleotides, in base pair, as indicated on the Pseudomonas genome site ([http://www.pseudomonas](http://www.pseudomonas/).com).
